# Water quality and residents' health: a survey by the self-assessed health method

**DOI:** 10.3389/fpubh.2025.1520354

**Published:** 2025-03-19

**Authors:** Jing Yan, Shuai Cui

**Affiliations:** ^1^Party School of the Shanxi Committee of the Chinese Communist Party, Shanxi Academy of Governance, Taiyuan, China; ^2^School of Marxism, Shanxi Normal University, Taiyuan, China

**Keywords:** water quality, water ecological pollution, self-assessed health rating, oprobit model, robustness check

## Abstract

**Introduction:**

Water is the source of life. The insufficient water resources and deteriorating water quality pose significant challenges to public health. This study investigates the impact of water quality on residents' self-assessed health rating using data from the 2016 China Genuine Progress indicator Survey. The analysis focuses on household cooking water sources (river/lake, well, tap, mineral/purified/filtered) and water pollution exposure in living or working environments.

**Methods:**

An ordered probit (oprobit) model was employed to analyze the relationship between water quality and residents' self-assessed health ratings, controlling for accessibility of medical services, individual lifestyles, and socio-demographic characteristics. The study also conducted heterogeneity analysis based on socioeconomic status and robustness checks using alternative dependent variables and estimation methods.

**Results:**

Results indicate that transitioning from river/lake water to safer sources-well, tap, and mineral/purified/filtered water-increases the probability of residents reporting self-assessed health ratings as “very good” by 7.9%, 10.4%, and 12.9%, while reducing the likelihood of “very bad or not very good” ratings by 7.2%, 9.4%, and 11.7%, respectively. Conversely, exposure to water pollution decreases the probability of “very good” health ratings by 2.4% and increases “very bad or not very good” ratings by 2.1%. The impact of cooking water quality on residents' health is more significant for lower socioeconomic status groups, while water pollution exposure affects higher socioeconomic status groups more. Robustness checks using hospitalization days as an alternative dependent variable and replacing oprobit with ologit/OLS models confirm these findings.

**Discussion:**

The study underscores the critical role of safe water access and ecological protection in enhancing public health. Policy recommendations include using and managing water resources strictly for holistic water security, maximizing the potential of China's revised Environmental Protection Laws, establishing a cross-agency coordination mechanism to tackle pollution sources, and improving medical services and fitness facilities to advance the “Healthy China” initiative.

## 1 Introduction

Labor force is one of the most active and important production factors, and also the source of material and social wealth creation. A healthy physique is the basic condition for achieving wealth accumulation. During China's National Health Conference in 2016, Chinese President Xi Jinping said that health is the foundation of career, family, reputation and wealth, so that human health should be placed on the strategic position of priority development. In the report of the 19th National Congress of the Communist Party of China published in 2017, President Xi Jinping further proposed the development strategy of “Healthy China”, considering human health as an important symbol of national prosperity. The report of the 20th National Congress in 2022 pointed out that we should promote the construction of “Healthy China”, improve people's wellbeing and living quality.

Water is the source of life. Water resource has become one of the crucial factors affecting human health. China's per capita water resource is lower than the world average level and the water ecological pollution is intensifying, meaning China facing the dual challenges of insufficient water quantity and poor water quality. On the one hand, water deficiency has become a serious problem. According to a report called “Water Scarcity” issued by the World Resource Institute, China accounts for about 20% of world population but only 7% water resource, hence ranking 109th in terms of the available freshwater resources per capita among all countries. Following the over-exploitation of water resource, China faces severe challenges such as reduced or broken surface runoff and the need to deepen water wells. On the other hand, water pollution has rendered freshwater unusable and endangered ecosystems. Simultaneously, the damaged water ecology environment has become a bottleneck restricting socio-economic development.

Rapid economic growth leads to environmental degradation, which in turn affects residents' health. Yang et al. (1) conclude that the substitution effect of economic growth on residents' health is greater than the income effect, so the residents' health level declines instead of rises. Health, as an important human capital, affects labor efficiency and household income, in turn has an impact on economic growth (2). Therefore, to promote the construction of Healthy China, we must effectively solve the problem of environmental pollution, such as the restoration of water ecology and the improvement of water quality. Based on the data of China Genuine Progress indicator Survey, taking self-assessed health rating as the dependent variable, the main source of household cooking water and the dichotomous variable of water pollution exposure in living or working place as the independent variables, this article establishes an oprobit model to analyze the impact of water quality on residents' health outcome while controlling for the accessibility of medical services, individual lifestyles as well as socio-demographic characteristics.

The marginal contributions of this study are as follows: (1) based on the new data from China Genuine Progress indicator Survey, which is different from the existing literature, (2) taking a more comprehensive and holistic health indicator, that is self-assessed health rating, which is equivalent to general health (GH) in the MOS 36-item Short Form Health Survey (SF-36), (3) with regard to the research object, this article discusses the impact of water quality on residents' health from a micro perspective, which is an important supplement to the existing literature, considering that the latter focusing on relevant research at the macro level.

## 2 Literature review

Health means that all physiological functions are normal, free from defects and diseases. It is the basic wellbeing pursued by mankind. As a kind of human capital, investing in health will yield the efficient time (3), subsequently promoting productivity and increasing economic growth rate (4). The indicators of health are derived from professional questionnaires, for example the Chinese Health Questionnaire (CHQ) (5) and the MOS 36-item Short Form Health Survey (SF-36) developed by the Health Institute of New England Medical Center in the USA (6). The former includes three factors, such as somatic symptoms, anxiety and depression. The latter includes eight dimensions of physical and mental health, namely general health (GH), physical functioning (PF), role-physical (RP), body pain (BP), vitality (VT), social functioning (SF), role-emotional (RE), mental health (MH) and health transition (HT). Additionally, health indicators used commonly also include mortality and morbidity rate, BMI or obesity among others.

Health is the result of multiple factors working together. From a specific perspective, the existing literature explores the influencing factors of health from the following aspects.

### 2.1 Accessibility of medical services

A healthy body requires the timely supply of nutrition, medical treatment and medicines, etc. Li and Yu (7) find that the diagnostic and treatment levels of village clinics are significantly positive correlated with villagers' physiological health, and the distance from village to the nearest medical facility has a significant impact on all SF-8 health indicators. The closer the distance is, the healthier the villagers will be. In addition, increasing medical insurance coverage rate, which means promoting the preventive medical service utilization, is helpful to human health (8). As an annotation, older adults with medical insurance have higher total health care expenditure, longer life expectancy, and better health status (9).

### 2.2 Individual lifestyles

A healthy lifestyle is necessary to maintain a good physical condition. A study about the United Kingdom finds that healthy lifestyles, such as eating breakfast, sleeping well, quitting smoking and drinking as well as exercising, help reduce the mortality rate and contribute to physical health (10). In an analysis about the causes of death in the United States in 2000, smoking, drinking, irrational diet and lack of exercise account for 38.2%, meaning the unhealthy lifestyle is a major cause of death (11). Additionally, the rising socio-economic status (12) and rapid urbanization (13) accelerate the decline in the health level of residents by changing individual lifestyles, such as an increase in high-fat diet and a decrease in physical activity.

### 2.3 Socio-demographic characteristics

Health is associated with gender (14), age (15) and marital status (7), etc. Generally, men are healthier than women because of male physical advantages. And individual's physical health level declines with age; the married group is healthier than others. Education attainment is positively correlated with the self-assessed health rating by improving working and economic conditions as well as obtaining more psychosocial resources (16). Based on survey data of the Wisconsin Longitudinal Study from 1957 to 2003, Herd (17) finds that education attainment contributes to individuals' physical health at an older age by improving cognitive abilities.

### 2.4 Ecological environment

Environmental health economics begins with the introduction of environmental pollution into the health production function by Cropper (18), Gerking and Stanley (19). The rapid economic development exerts great pressure on the environment, especially causing air and water pollution, which directly affect residents' health (20). Studies based on the provincial panel data in China find that environmental pollution has a significant negative impact on public health (21) and that further contributes to the health care expenditures (22). Chen and He (23) introduce the impact of environmental pollution on health using an overlapping-generation model, find that public health is negatively related to the intensity of pollutant emissions and positively related to the investment in reducing pollutant emissions. The more the emissions reduction investment, the less the pollutant stock, and the better the public health will be.

Most studies about the impact of ecological environment on public health focuses on air and water quality at a macro perspective. In terms of the impact of air quality on public health, Chay and Greenstone (24) take the United States between 1981 and 1982 as an example and find that the infant mortality rate decreases by 0.35% for every 1% reduction in the total suspended particulates (TSP) caused by the economic recession. Mead and Brajer (25) take Chinese children as the research object to quantify the cost of childhood morbidity at the municipal level, and find that the extensive use of fossil fuels has significantly increased the incidence of cardiovascular, cerebrovascular, and respiratory diseases among children. Using panel data of China's prefecture-level cities, Chen et al. (26) find that air pollution increases the mortality rate induced by respiratory diseases and lung cancer. Qi and Lu (27) estimate the economic burden of deteriorating health caused by air pollution in 112 key cities of China from 2003 to 2010, and find that the burden is heavier in regions with underdeveloped economy. Conversely, the implementation of environment-friendly policies by the government contributes to the reduction of health costs (28).

The quality of water is directly related to residents' physical health. With the acceleration of industrialization and urbanization, the problem of water pollution has become increasingly severe, triggering extensive academic research on the relationship between water quality and residents' health. Contaminants in water sources pose significant risks, contributing to acute and chronic health conditions. The existing research on the relationship between water quality and health outcome focuses on heavy metals, chemical contaminants, and emerging pollutants. (1) Heavy metals. Heavy metals such as lead and chromium accumulate in water. After entering the human body through drinking water, they can damage the nervous system, kidneys, bones, etc. Two studies, based on city-level data in the USA from 1900 to 1920 (29) and Massachusetts data in 1900 (30), find that the use of lead water pipes increases the amount of lead in drinking water, subsequently promoting the increase in infant and child mortality rates. In India, those consuming heavy metals contaminated water especially with Cr are identified to be highly prone to cancer risk (31). (2) Chemical contamination. Chemical pollutants, including nitrates, pesticides, and industrial chemicals, are associated with chronic health issues. Pesticides, as an organic pollutant, are carcinogenic and mutagenic. The consumption of water contaminated with pesticides increases the risk of cancer in residents. In India, compared to villages irrigated with normal water, those irrigated with wastewater have a higher incidence rate (32). In Indonesia, many water sources are contaminated by various pollutants, which force residents to rely on unimproved water sources, such as river water, for their daily use. As a result, the incidence of waterborne diseases such as diarrhea has significantly increased (33). In many areas of the world, nitrate contamination from agricultural runoff correlates with methemoglobinemia in infants and potential colorectal cancer risk. The infiltration of agricultural fertilizers causes nitrate pollution to groundwater and long term consumption of water with excessive nitrate levels significantly increases the risk of colorectal cancer (34). A Danish study finds that higher nitrate level in drinking water is correlated with preterm births and congenital anomalies (35). (3) Emerging pollutants. In addition to chemical and heavy metals contaminants, the issue of antibiotic residues in the water environment has increasingly drawn attention. The World Health Organization emphasized the hazards of antibiotics in water. With the widespread use of antibiotics in fields such as healthcare and livestock farming, a large amount of incompletely metabolized antibiotics enter the water environment through various channels. Residual antibiotics in water may not only promote the emergence and spread of drug-resistant bacteria, gradually rendering originally effective antibiotics ineffective in treating diseases and threatening global public health security. They can also directly enter the human body through drinking water, disrupting the normal balance of the human micro-biota, affecting the immune system and metabolic functions, thus potentially causing long-term damage to residents' health.

In summary, the existing literature examines health issues mainly from the perspective of accessible medical services, individual lifestyles, socio-demographic characteristics as well as ecological environment, etc. In terms of ecological environment, access to safe drinking and cooking water is a cornerstone of public health, yet water quality remains a critical issue. Regarding the impact of water quality on health, most studies focus on underdeveloped countries or developed countries from earlier years, discussing the relationship between the excessive harmful substances in water and the incidence of certain diseases. Contaminants in water sources pose significant risks, contributing to acute and chronic health conditions. According to the viewpoints of Arbaz, Rahul and Chatterjee, the reduced morbidity as well as mortality rate of developing countries in recent years is attribute to the generation of safe and adequate consumable water (36). In addition, the existing literature analyzes the impact of water quality on residents' health at the provincial or municipal level. The only few studies about China focus on the heavily polluted Huai River Basin, where water pollution promotes the rise of cancer incidence rate (37). The death rate of digestive system cancer increases by 9.7% for one-grade decrease in the water quality of Huai River (38). These studies regard water quality of Huai River Basin as the key explanatory variable at the macro level and residents' health as the dependent variable at the micro level, which leads to a mismatch between the data level and the theoretical analysis object. Finally, different groups face varying levels of water quality. Generally speaking, groups with lower socioeconomic status face poorer water quality. A report highlights that a staggering two billion people don't have access to safely managed drinking water around the world in 2020. Among them, 771 million individuals lack basic drinking water, with the majority being in sub-Saharan Africa. In particular, compared to urban residents in sub-Saharan Africa, those in rural areas face a more acute water access problem. Take the Democratic Republic of Congo as an example: only 22% of the rural population has access to the basic drinking water services, while this figure reaches 75% for the urban population (39).

Different from the existing literature, this article discusses the impact of household cooking water quality and living or working environment with water pollution on China residents' self-assessed health rating at a micro-individual perspective by establishing the Oprobit model based on China Genuine Progress indicator Survey. At the same time, the model controls for the accessibility of medical services, individual lifestyle and socio-demographic characteristics. Considering that most of the relevant literature analyzes the correlation of water quality and residents' health based on macro-level data at the regional level, this article provides a beneficial supplement to the existing literature.

## 3 Variables and model

### 3.1 Data source

The variables in this article are from the China Genuine Progress indicator Survey (CGPiS) hosted by Beijing Normal University in 2016. The survey is conducted in Beijing and Chengdu and involves many aspects of information, such as community characteristics, public service facilities, environmental sanitation, and individual socio-demographic characteristics, employment and income, health, volunteerism, values, and so on. More importantly, the database contains both subjective/objective health indicators and water quality-related variables. This article analyzes the impact of water quality on residents' health. After excluding missing values of variables, this article has 3,612 observations in the sample.

### 3.2 Dependent variable

The dependent variable is the self-assessed health rating, with options 1–5 assigning from “very bad” to “very good”, respectively. Because there are only 60 respondents who reporting “very bad”, we combine two options of “very bad” and “not very good” so that the self-assessed health rating ranges from “very bad or not very good” to “very good” with values of 1–4. As shown in Table 1, both selections of “fair” and “relatively good” account for the largest proportion at 76.22% , while the percentages of respondents who choose “very good” and “very bad or not very good” only account for 12.62% and 11.16%, respectively.

**Table 1:** The distribution of residents' self-assessed health rating.

**Health**	**Frequency**	**Percentage**
1 Very bad or not very good	403	11.16%
2 Fair	1,452	40.20%
3 Relatively good	1,301	36.02%
4 Very good	456	12.62%
Total	3,612	100.00%

Table 2 shows the definition and description of all variables. The average value of individual self-assessed health rating is 2.501, which means lots of people believe that their bodies are relatively healthy.

**Table 2:** Descriptive statistics (observations = 3,612).

**Category**	**Variable**	**Definition**	**Mean**	**Standard deviation**	**Min**.	**Max**.
Dependent variable	Health	Self-assessed health rating, from “very bad or not very good” to “very good” with values of 1–4	2.501	0.852	1	4
Key independent variables	Main source of cooking water used by residents				
	Cooking water 1	River and lake water= 1, otherwise= 0	0.010	0.101	0	1
	Cooking water 2	Well water = 1, otherwise = 0	0.150	0.357	0	1
	Cooking water 3	Tap water = 1, otherwise = 0	0.825	0.380	0	1
	Cooking water 4	Mineral/purified/filtered water = 1, otherwise = 0	0.015	0.120	0	1
	Water pollution	Water pollution in living or working place, Yes = 1, No = 0	0.281	0.450	0	1
Accessibility of medical services	Hospital	Availability of a hospital in the community, Yes=1, No=0	0.257	0.437	0	1
	Medical point	Availability of a medical point (healthcare station, clinic, pharmacy) in the community, Yes=1, No=0	0.885	0.319	0	1
Individual lifestyles	Exercise place	Less than 20 min spent on walking from residential area to activity venues such as plazas, parks, playgrounds, gyms, swimming pools, etc., Yes = 1, No = 0	0.836	0.370	0	1
	Exercise time	Time spent on exercising (hours/week)	4.405	5.545	0	28
	Smoke	Number of cigarettes smoked per day	2.998	6.297	0	20
Socio-demographic characteristics	Male	Male=1,Female=0	0.454	0.498	0	1
	Age	Years old	46.460	13.958	16	70
	Marital status					
	Marriage 1	Unmarried = 1, otherwise = 0	0.111	0.314	0	1
	Marriage 2	Married = 1, otherwise = 0	0.831	0.374	0	1
	Marriage 3	Divorced or widowed = 1, otherwise = 0	0.058	0.234	0	1
	Eduyear	Years of receiving education	10.044	4.128	0	21
Urban/rural	Urban	Urban = 1, Rural = 0	0.793	0.405	0	1

### 3.3 Independent variables

The quality of household cooking water is measured by the selection of “the main source of cooking water used by residents”, which mainly includes river and lake water, well water, tap water and mineral/purified/filtered water. The four options account for 1%, 15%, 82.5%, and 1.5%, respectively. Although the vast majority of communities use tap water, there is still small number of residents who use water from river, lake or well to cook. The residents' health will be deteriorated consequently when polluted water is discharged into rivers and lakes, or infused into the groundwater.

Water pollution is represented by “the dichotomous variable of water pollution exposure in living or working place”. From the perspective of individual sample, 28.1% of the respondents believe that water pollution exists in their residential or working places (Table 2). In fact, among all of 79 community samples, 14% of community leaders believe that water pollution has the greatest negative impact on residents' daily life.

### 3.4 Control variables

According to the existing literature, this article control for three kinds of variables including accessible medical services, individual lifestyles, and socio-demographic characteristics. (1) The accessibility of medical services refers to the timeliness and convenience of obtaining medicines, nutrition and health guidance. In the sample, respondents reporting that there is at least one hospital or medical point in the community account for 25.7% and 88.5%, respectively. Both of them provide residents with convenient conditions to enjoy medical service, subsequently prevent the occurrence of serious diseases. (2) Individual lifestyle refers to the activities of daily life, including the utilization of leisure time. Generally speaking, the distance between residential area and activity venues is an important factor determining the frequency and duration of residents' participation in exercise. According to the survey data, there are 83.6% of the respondents whose distance from their residence to activity venues such as plazas, parks, playgrounds, gyms, swimming pools, etc. is within a 20-min walk. In addition, all respondents spend about 4.405 h on exercising every week, with a maximum value of 28 h. For the full sample, the average number of cigarettes smoked per day is about 3. (3) For socio-demographic characteristics, 45.4% of the respondents are male; all respondents are aged between 16 and 70, with an average age of 46.5 years old. Regarding the marital status, the unmarried, married, divorced or widowed account for 11.1%, 83.1% and 5.8%, respectively; the average years of receiving education for all respondents is 10 years approximately at the stage of having completed junior high school education but not having completed senior high school education. (4) Finally, the respondents living urban areas account for 79.3%.

### 3.5 Model selection

The empirical model set up in this article is:


(1) $$\begin{array}{l} health=\beta \text{X}+\mu \end{array}$$

Where **X** denotes the influencing factors of individual self-assessed health rating, including household cooking water quality, the dichotomous variable of water pollution exposure at one's residential or working environment, as well as the accessibility of medical services, individual lifestyles, socio-demographic characteristics and urban-rural dichotomous variable.

The dependent variable *health* refer to the self-assessed health rating with four options “very bad or not very good”, “fair”, “relatively good” and “very good” of values 1, 2, 3 and 4, respectively. We establish an oprobit model because the self-assessed health rating is an ordered discrete variable. In fact, the physical health is a continuous variable, which is not limited to the above four levels. When the respondent's self-assessed health (health^*^) is below a certain threshold C_1_, it is reported as “very bad or not very good”; when it is above the threshold C_1_ but below C_2_, it is reported as “fair”; when it is above the threshold C_2_ but below C_3_, it is reported as “relatively good”; when it is above the threshold C_3_, it is reported as “very good”, i.e.,


(2) $$\{\begin{array}{ll} health=1,\;if\;health^{*}\;\leq \;C_{1} & \;\\ health=2,\;if\;C_{1}\;<\;health^{*}\;\leq \;C_{2} & \;\\ health=3,\;if\;C_{2}\;<\;health^{*}\;\leq \;C_{3} & \;\\ health=4,\;if\;health^{*}\;>\;C_{3} & \; \end{array}\;$$

Where C_1_, C_2_, C_3_ are referred to as cut-off points, and C_1_ < C_2_ < C_3_.

Assuming that φ(•) is the cumulative distribution function of μ, which is the random error term and follows the standard normal distribution, the conditional probability of *health* taking 1, 2, 3 and 4 can be expressed as (40):


(3) $$\{\begin{array}{ll} P\;(health=1)\;=\;\varphi \;(C_{1}\;-\;X\beta ) & \;\\ P\;(health=2)\;=\;\varphi \;(C_{2}\;-\;X\beta )\;-\;\varphi \;(C_{1}\;-\;X\beta )\; & \;\\ P\;(health=3)\;=\varphi \;(C_{3}\;-X\beta )\;-\;\varphi \;(C_{2}\;-\;X\beta \;) & \;\\ P\;(health=4)\;=\;1-\varphi \;(C_{3}\;-\;X\beta )\; & \; \end{array}$$

The sum of the above probabilities is 1. β represents the parameter vector to be estimated by constructing a likelihood function for each response and using the maximum likelihood estimation (MLE) method.

## 4 Results

### 4.1 Regression analysis results

Table 3 shows the benchmark regression results. For Models (1)–(6), the method of adding variables stepwise is used, where model (1) only includes the main source of water used for household cooking, model (2) adds the dichotomous variable of water pollution exposure, and models (3)–(5) further control for the accessibility of medical services, individual lifestyles and socio-demographic characteristics. Model (6) controls for the urban-rural dichotomous variable.

**Table 3:** The influencing factors of Chinese residents' self-assessed health rating from oprobit regression.

	**Dependent variable** = **self–assessed health rating**					
**Model**	**(1)**	**(2)**	**(3)**	**(4)**	**(5)**	**(6)**
**Cooking water (reference group** = **cooking water 1)**						
Cooking water 2	0.569^***^	0.562^***^	0.545^***^	0.432^**^	0.397^**^	0.394^**^
	(0.186)	(0.186)	(0.187)	(0.190)	(0.190)	(0.191)
Cooking water 3	0.764^***^	0.759^***^	0.744^***^	0.622^***^	0.521^***^	0.517^***^
	(0.181)	(0.181)	(0.182)	(0.185)	(0.186)	(0.188)
Cooking water 4	0.807^***^	0.820^***^	0.821^***^	0.702^***^	0.645^***^	0.640^***^
	(0.233)	(0.233)	(0.233)	(0.236)	(0.237)	(0.239)
Water pollution		−0.108^***^	−0.108^***^	−0.108^***^	−0.117^***^	−0.117^***^
		(0.040)	(0.040)	(0.040)	(0.040)	(0.040)
Hospital			0.095^**^	0.091^**^	0.086^**^	0.085^**^
			(0.041)	(0.042)	(0.042)	(0.042)
Medical point			0.076	0.059	0.039	0.038
			(0.057)	(0.058)	(0.058)	(0.058)
Exercise place				0.141^***^	0.084	0.083
				(0.051)	(0.052)	(0.053)
Exercise time				0.010^***^	0.014^***^	0.014^***^
				(0.003)	(0.003)	(0.003)
Smoke				0.004	0.002	0.002
				(0.003)	(0.003)	(0.003)
Male					0.110^***^	0.110^***^
					(0.041)	(0.041)
Age					−0.006^***^	−0.006^***^
					(0.002)	(0.002)
**Marital status(Reference group**=**Marriage 3)**						
Marriage 1					0.277^***^	0.277^***^
					(0.102)	(0.102)
Marriage 2					0.075	0.075
					(0.078)	(0.078)
Eduyear					0.010^**^	0.010^*^
					(0.005)	(0.005)
Urban						0.008
						(0.050)
Observation	3,612	3,612	3,612	3,612	3,612	3,612
Pseudo R-squared	0.004	0.004	0.005	0.008	0.017	0.017

^*^p < 0.1, ^**^p < 0.05, ^***^p < 0.01. The values in parentheses are standard errors.

Regarding river and lake water as the reference group, the coefficients of well, tap and mineral/purified/filtered water as the main source for household cooking are significantly positive at the usual statistical level. This indicates that the safe and clean water source contributes to residents' physical health. The coefficient of water pollution exposure is significantly negative at the statistical level of 1%, which means that respondents who reside or work in place where there is water pollution have a lower self-assessed health rating.

In terms of access to medical services residents living in the community with at least one hospital have a higher self-assessed health rating. Because community hospitals help promote residents' physical health by improving the accessibility of medical services, such as healthcare, nutrition knowledge, etc. However, the presence of a medical point in the community affects the self-assessed health rating insignificantly.

With regard to individual lifestyles the distance between residential area and activity venues, such as plazas, parks, playgrounds, gyms, swimming pools, etc., within a 20 min walk has a positive but not significant impact on residents' self-assessed health rating. The self-assessed health rating is significantly positive correlated with the average hours spent on physical activity per week, which means doing exercise is beneficial to health. After adding socio-demographic characteristics, the coefficient of exercise duration becomes larger, while that of the distance from residential area to activity venues become no longer significant, indicating that doing exercise not activity venues is helpful to improve residents' health. If you don't exercise, building more venues at the doorstep will be useless. Contrastingly, it is not confirmed in this study that “smoking is harmful to health”. Because 77% of the respondents are non-smokers, and the average number of cigarettes smoked daily is about 3 for the full sample, which is relatively less harmful to health.

In the aspect of socio-demographic characteristics the self-assessed health rating is higher for males than females. On the one hand, men have a comparative advantage in physiological aspect; on the other hand, females receive lower health return from education and income than males. The self-assessed health rating decreases with age, that is physical function has a tendency to decay with the increasing age, which is consistent with the actual situation. The self-assessed health rating of unmarried respondents is relatively higher, because they are younger and in better physical condition, considering that the age of our sample is between 16 and 70 years old. The coefficient of receiving education years is significantly positive at the level of 1%, indicating that the higher the level of receiving education, the higher the individual's self-assessed health rating, which is consistent with existing studies.

Finally, the coefficient of the urban-rural binary variable is not significant. That is to say, living in urban or rural areas has no significant impact on residents' health.

### 4.2 Marginal effects

The result reported by oprobit model can be used to illustrate the correlation between influencing factors and self-assessed health rating. However, the coefficient values are not marginal effects and cannot represent specific quantitative relationships.

Taking model (6) in Table 3 as an example, Table 4 reports the marginal effects of some influencing factors at their mean values. According to the safety of water source, mineral/purified/filtered water is the best, followed by tap water, well water is ranked third, river and lake water is the worst. The change in household cooking water source from river and lake to well, tap, mineral/purified/filtered water increases the probability of respondents' self-assessed health rating as “very good” by 7.9%, 10.4%, and 12.9%, and reduces the probability of reporting “very bad or not very good” by 7.2%, 9.4% and 11.7%, respectively. Additionally, both change magnitudes in two kinds of probabilities exhibit an increasing trend. And the absolute values of z are all >2, indicating that the coefficients of marginal effects are significant at the 1% statistical level. Therefore, the safer the water source, the better the water quality, the higher the individual's self-assessed health rating. On the contrary, the worse the cooking water quality, the lower the self-assessed health rating will be.

**Table 4:** The marginal effect of some influencing factors affecting Chinese residents' self-assessed health rating.

	**Self-assessed health rating**=**4**		**Self-assessed health rating**=**1**	
	**Marginal effect**	***z***	**Marginal effect**	***z***
Cooking water 2	0.079	2.060	−0.072	−2.060
Cooking water 3	0.104	2.740	−0.094	−2.740
Cooking water 4	0.129	2.670	−0.117	−2.670
Water pollution	−0.024	−2.930	0.021	2.930
Hospital	0.017	2.010	−0.016	−2.010
Exercise time	0.003	4.070	−0.003	−4.060

The marginal effects are corresponding to the oprobit results of model (6) in Table 3.

With regard to the dichotomous variable of water pollution exposure in living or working place, if it changes from “without water pollution” to “with water pollution”, the probability of respondents' self-assessed health rating as “very good” decreases by 2.4%, and the probability of reporting “very bad or not very good” increases by 2.1%. It can be seen that protecting the water ecological environment is very important for residents' health.

Table 3 shows that community hospital and weekly exercise time affect individuals' self-assessed health rating significantly. According to Table 4, the presence of community hospital increases the probability of respondent's reporting “very good” by 1.7% and decreases the probability of reporting “very bad or not very good” by 1.6%. If the average time spent on exercise activity per week increases by 1 h, the probabilities of reporting “very good” and “very bad or not very good” rise and decrease with the same magnitude by 0.3%.

### 4.3 Heterogeneity analysis

Generally speaking, respondents receiving longer years of education have more decent jobs, higher income and consumption levels, and better hygiene conditions, while those without receiving education or with a lower education level lagged behind. Therefore, the variable of residents' receiving education years, to some extent, can replace socio-economic status. That is to say, respondents receiving higher education level also have a higher socioeconomic status.

In benchmark regression, we conducted model by gradually adding control variables. Even after controlling for all variables, the impact of education years on health remained significant, indicating that respondents with higher socioeconomic status tend to report higher self-assessed health ratings. Considering that individuals with junior high school education constitute approximately 30% in our sample, we stratify respondents into two subgroups: a lower-education subsample (educational attainment < 8 years) and a higher-education subsample (educational attainment more than or equal to 8 years) according to whether junior high school education has been completed or not.

The subsample analysis results are presented in Table 5. For the lower-education subsample, the impact of cooking water quality on self-assessed health rating remains consistent with the benchmark regression result. But the coefficient of mineral/purified/filtered water as the main source for household cooking is not significant at the usual statistical level. Maybe because only a very small number of residents use mineral/purified/filtered water for cooking, even in the total sample, only 1.5% of households use mineral/purified/filtered water as their main source for daily cooking. And these households are more likely to belong to the sub-sample with higher education level. In contrast, all coefficients of cooking water quality become statistically insignificant for the higher-education subsample. It will be seen from this that socioeconomically disadvantaged groups (lower-education subsample) face a more acute water access challenge. Consequently, the improvement in water resource for household cooking enhances self-assessed health outcomes significantly within this subgroup.

**Table 5:** Heterogeneity analysis: subsamples differentiated by educational level.

	**Dependent variable** = **self-assessed health rating**					
**Model**	**(1)**	**(2)**	**(3)**	**(4)**	**(5)**	**(6)**
Sub-sample	Eduyear < 8	Eduyear ≥ 8	Eduyear < 8	Eduyear ≥ 8	Eduyear < 8	Eduyear ≥ 8
Regression method	Oprobit	Oprobit	Ologit	Ologit	Ols	Ols
**Cooking water (reference group** = **cooking water 1)**						
Cooking water 2	0.560^**^	0.129	0.852^*^	0.236	0.466^**^	0.087
	(0.273)	(0.276)	(0.449)	(0.514)	(0.228)	(0.207)
Cooking water 3	0.606^**^	0.282	0.975^**^	0.515	0.498^**^	0.201
	(0.267)	(0.272)	(0.437)	(0.508)	(0.223)	(0.204)
Cooking water 4	0.512	0.423	0.961	0.765	0.457	0.309
	(0.489)	(0.315)	(0.875)	(0.576)	(0.418)	(0.237)
Water pollution	0.081	−0.160^***^	0.123	−0.296^***^	0.059	−0.123^***^
	(0.095)	(0.044)	(0.163)	(0.076)	(0.082)	(0.033)
Accessibility of medical services	Controlled	Controlled	Controlled	Controlled	Controlled	Controlled
Individual lifestyle	Controlled	Controlled	Controlled	Controlled	Controlled	Controlled
Socio-demographic characteristics	Controlled	Controlled	Controlled	Controlled	Controlled	Controlled
Urban or rural	Controlled	Controlled	Controlled	Controlled	Controlled	Controlled
Constant					1.936^***^	2.318^***^
					(0.355)	(0.230)
Cut_cons	Yes	Yes	Yes	Yes		
Observation	678	2,934	678	2,934	678	2,934
(Pseudo) Adj R-squared	0.018	0.016	0.017	0.016	0.025	0.034

(1)^*^p < 0.1, ^**^p < 0.05, ^***^p < 0.01. The values in parentheses are standard errors.

(2)The regression results of the accessibility of medical services, individual lifestyles and socio-demographic characteristics are similar to model (6) in Table 3. There will not be repeated.

For water pollution exposure, the coefficient for living/working in areas with water pollution shows a significantly negative correlation with residents' self-assessed health rating for the higher-education subsample. No significant correlation is observed in the lower-education subsample. That is to say, the higher-education subgroup demonstrates greater sensitivity to surrounding environmental condition, maybe due to their stronger awareness of ecological crisis. Conversely, the lower-education subgroup prioritizes immediate household-level water quality improvements over region-level environmental concerns.

### 4.4 Robustness check

**Figure 1:**
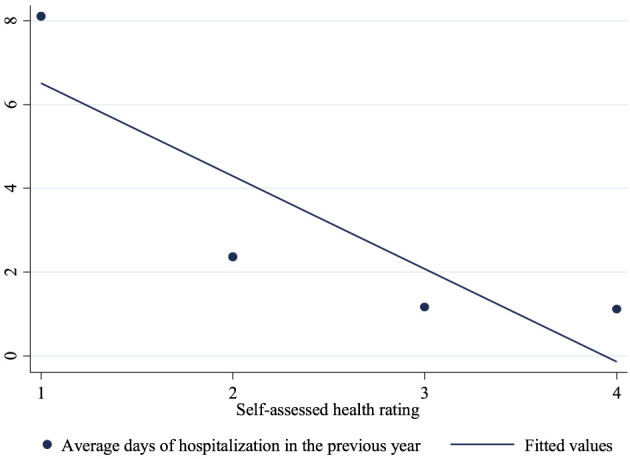
The negative correlation between average days of hospitalization and self-assessed health rating.

To test the robustness of the impact of cooking water quality and water pollution exposure on public health, we further use “days of hospitalization in the previous year” as the dependent variable to recalculate the model. To provide a more intuitive characterization of the relationship between the two variables, we calculate the mean length of hospital stay corresponding to each value of self-assessed health rating. Respondents with self-assessed health rating of 1 have the average hospitalization duration of 8.1 days. Similarly, those with self-assessed health rating of 2, 3, and 4 show average hospitalization durations of 2.4, 1.2, and 1.1 days, respectively. Based on these calculations, we plot a fitted line illustrating the relationship between average hospitalization duration and self-assessed health rating, as shown in Figure 1. A negative correlation is observed between the objective hospitalization duration and the subjective self-assessed health rating. Furthermore, the correlation coefficient between “days of hospitalization in the previous year” and self-assessed health rating is −0.1725 and significant at the statistical level of 1%.

Considering that the new dependent variable, “days of hospitalization in the previous year”, is count data, Table 6 shows the Poisson regression result. Using river and lake water as the reference group, the coefficients of well, tap and mineral/purified/filtered water as main source for household cooking are significantly negative at the usual statistical level in models (1)–(6), except for the coefficients for well water in model (5) and (6). This indicates that the safer the cooking water source, the better the water quality, the fewer the hospital stay and the healthier the residents' physical body. The coefficient of water pollution is significantly positive at the level of 1%, indicating that respondents who reside or work in areas with water pollution exposure have poorer physical health and longer hospital stays. This is consistent with the previous result in Table 3.

**Table 6:** Robustness check from poisson regression: using hospitalization days as the dependent variable.

	**Dependent variable** = **days of hospitalization in the previous year**					
**Model**	**(1)**	**(2)**	**(3)**	**(4)**	**(5)**	**(6)**
**Cooking water (reference group** = **cooking water 1)**						
Cooking water 2	−0.429^***^ (0.077)	−0.418^***^ (0.077)	−0.412^***^ (0.078)	−0.203^**^ (0.079)	−0.102 (0.08)	−0.040 (0.08)
Cooking water 3	−0.847^***^ (0.074)	−0.840^***^ (0.074)	−0.848^***^ (0.075)	−0.509^***^ (0.077)	−0.226^***^ (0.077)	−0.140^*^ (0.078)
Cooking water 4	−1.611^***^ (0.153)	−1.628^***^ (0.153)	−1.627^***^ (0.153)	−1.131^***^ (0.155)	−0.953^***^ (0.155)	−0.847^***^ (0.156)
Water pollution		0.142^***^ (0.023)	0.139^***^ (0.023)	0.166^***^ (0.023)	0.204^***^ (0.023)	0.202^***^ (0.023)
Accessibility of medical services			Controlled	Controlled	Controlled	Controlled
Individual lifestyle				Controlled	Controlled	Controlled
Socio-demographic characteristics					Controlled	Controlled
Urban or rural						Controlled
Constant	1.648^***^ (0.073)	1.598^***^ (0.074)	1.418^***^ (0.082)	1.208^***^ (0.083)	−0.056 (0.115)	−0.090 (0.116)
Observation	3,609	3,609	3,609	3,609	3,609	3,609
Pseudo R-squared	0.009	0.009	0.011	0.040	0.076	0.077

(1)^*^p < 0.1, ^**^p < 0.05, ^***^p < 0.01. The values in parentheses are standard errors.

(2) The regression results of the accessibility of medical services, individual lifestyles and socio-demographic characteristics are similar to those in Table 3. There will not be repeated.

Table 7 reports the results of Ologit and Ols regressions to tests the model robustness. The direction and significance level of coefficients for each variable are highly consistent with the previous text. Therefore, they will not be repeated.

**Table 7:** Robustness check from Ologit and Ols regressions.

	**Dependent variable** = **self-assessed health rating**	
**Model**	**(1)**	**(2)**
Regression method	Ologit	Ols
**Cooking water (reference group** = **cooking water 1)**		
Cooking water 2	0.648^*^ (0.335)	0.291^**^ (0.146)
Cooking water 3	0.887^***^ (0.329)	0.383^***^ (0.144)
Cooking water 4	1.135^***^ (0.416)	0.483^***^ (0.184)
Water pollution	−0.224^***^ (0.069)	−0.095^***^ (0.031)
Hospital	0.150^**^ (0.073)	0.065^**^ (0.033)
Medical point	0.044 (0.101)	0.028 (0.045)
Exercise place	0.183^*^ (0.094)	0.064 (0.041)
Exercise time	0.026^***^ (0.006)	0.011^***^ (0.003)
Smoke	0.003 (0.006)	0.001 (0.003)
Male	0.163^**^ (0.071)	0.083^**^ (0.032)
Age	−0.010^***^ (0.003)	−0.005^***^ (0.001)
**Marital status (Reference group** = **marriage 3)**		
Marriage 1	0.468^***^ (0.177)	0.219^***^ (0.080)
Marriage 2	0.126 (0.136)	0.059 (0.061)
Eduyear	0.019^**^ (0.009)	0.008^*^ (0.004)
Urban	−0.003 (0.088)	0.004 (0.039)
Constant		2.041^***^ (0.179)
Cut_cons	Yes	
Observation	3,612	3,612
(Pseudo) Adj R-squared	0.017	0.040

^*^p < 0.1, ^**^p < 0.05, ^***^p < 0.01. The values in parentheses are standard errors.

## 5 Conclusion and discussion

The extensive economic growth at the cost of the ecological environment damage has further led to health crises. This article uses the oprobit model to explore the influences of household cooking water quality and water pollution exposure on residents' self-assessed health rating from a micro individual perspective, based on China Genuine Progress indicator Survey data in 2016. For the household cooking water, there are four main water sources, such as river and lake water, well water, tap water and mineral/purified/filtered water. When the main source of cooking water changes from river and lake water to well, tap, or mineral/purified/filtered water, the probability of respondents' self-assessed health rating as “very good” increases by 7.9%, 10.4%, and 12.9%, and the probability of reporting “very bad or not very good” decreases by 7.2%, 9.4%, and 11.7%, respectively. The magnitude of the change in both probabilities shows an increasing trend, indicating that the safe and clean cooking water contributes to residents' self-assessed health rating. And groups with lower socioeconomic status face a more severe water access problem and are more sensitive to the improvement in household cooking and drinking water quality. In fact, China has a population of 1.4 billion but limited water resource, and both the total amount of water resources and the per capita water resources have been decreasing over the past 20 years based on the Statistical Yearbook compiled by National Bureau of Statistics of China in 2024. Especially vulnerable groups, for example population in rural area and northwest region, face more severe water safety issues. As a result, we should use and manage water resources strictly and meticulously. In view of this, we need to pay more attention to the paradigm shift from quantity-focused water exploitation to quality-driven and sustainable allocation, which is helpful to reduce water intensity (water use per unit of GDP), raise the proportion of surface water rated Grade III or higher (suitable for human contact), and provide vouchers for low-income households to install water filters to ensure cooking/drinking water safety. Water is not only a resource but also a foundation of human dignity, China can model a pathway for sustainable development that prioritizes the most vulnerable—ensuring safe water access becomes a universal reality, not an aspirational goal.

According to our findings, the respondents' self-assessed health rating is significantly negative correlated with water pollution exposure, meaning that individuals living or working in areas affected by water pollution are more likely to report a lower self-assessed health rating. When the area where residents live or work changes from “without water pollution” to “with water pollution”, the probability of respondents' self-assessed health rating as “very good” decreases by 2.4% and the probability of reporting “very bad or not very good” increases by 2.1%. This result becomes more significant statistically for groups with higher socioeconomic status. Therefore, we should maximize the potential of China's revised Environmental Protection Laws (2023 amendment) and Water Pollution Prevention and Control Law, such as strengthen the punishment for illegal emissions, implement real-time pollution monitoring for key industries, forcing polluters to fund river restoration, and expand public participation rights to establish a situation of nationwide supervision. Considering that the water ecology is an important component of the ecological environment shared by all humans, it is necessary to build a cross-agency coordination mechanism, establish interdepartmental task forces (environmental bureaus, health ministries, agricultural departments) to tackle pollution sources holistically. Specifically, government departments play a leading role, enterprises and residents respond actively and participate in water ecological environment protection, subsequently promote the residents' physical health, reduce days of hospitalization and improve residents' self-assessed health rating.

These three factors, the accessibility of medical services, individual lifestyles and socio-demographic characteristics, have significant influences on residents' self-assessed health rating. In terms of individual socio-demographic characteristics, male, young, unmarried and more educated respondents are more likely to report a higher self-assessed health rating. Regarding the accessibility of medical services, the presence of a hospital in the community is conducive to residents' self-assessed health rating by improving the accessibility of medical services, while whether there is a medical point in the community or not has no significant impact on public health. Maybe because the community hospital provides residents with an easy accessibility of medical services, subsequently help to curb the occurrence of serious illnesses in time. Regarding individual lifestyle, after controlling for socio-demographic characteristics, the coefficient of whether there is an exercise facility near the residential area is positive but insignificant, while the coefficient of exercise time is significantly positive at the usual statistical level. Maybe because the construction of activity venues in the half-hour living circle provides residents with convenient conditions for leisure and fitness, consequently increases the frequency and prolongs the time of exercise. Therefore, the government should increase financial expenditure for the construction of grass-root medical facilities and public activity venues, considering that both of them are helpful to promote residents' physical health by improving grass-root healthcare service and increasing residents' participation rate in exercising.

This article uses dataset different from the existing literature, conducts a one-to-one matching between water quality and residents' health, thereby avoiding the mismatch between the data level and the research object. As a more comprehensive and holistic health indicator, the self-assessed health rating is a subjective variable. So this article replaces the dependent variable with the objective indicator naming “days of hospitalization in the previous year” to test model robustness. And the results are consistent. In terms of methodology, this article further uses ologit and ols methods to recalculate the model and arrives at similar results. However, since our sample is derived from micro-level survey data collected in only two cities in 2016, it has limitations in geographical and temporal representativeness. Therefore, the promotion of the conclusion needs to be cautious.

In future studies, we will distinguish between subjective and objective indicators of residents' health, combine water quality data re-leased by authoritative departments with micro-survey datasets including more representative urban and rural areas, and use Hierarchical Linear Modeling (HLM) to conduct multi-level research. In addition, we will explore the intrinsic mechanisms by which water quality affects residents' health, and use time-series data to analyze the long-term trend in the relationship between water quality and residents' health in the context of evolving environmental and policy landscapes. Furthermore, we will design experiments including instrumental variables for water quality and prioritize causal analysis as an important research direction.

## Data Availability

Publicly available datasets were analyzed in this study. This data can be found here: https://chinaiid.bnu.edu.cn/yjpt/zgzsjbwgtcyyjzx/cgpis/index.html.
